# Test-retest reliability of the computer-assisted DIA-X-5 interview for mental disorders

**DOI:** 10.1186/s12888-020-02653-6

**Published:** 2020-06-05

**Authors:** Jana Hoyer, Catharina Voss, Jens Strehle, John Venz, Lars Pieper, Hans-Ulrich Wittchen, Stefan Ehrlich, Katja Beesdo-Baum

**Affiliations:** 1 Institute of Clinical Psychology and Psychotherapy, Technische Universität Dresden, Dresden, Germany; 2 Behavioral Epidemiology, Faculty of Psychology, Technische Universität Dresden, Dresden, Germany; 3 Center for Clinical Epidemiology and Longitudinal Studies, Technische Universität Dresden, Dresden, Germany; 4 Department of Psychiatry & Psychotherapy, Ludwig-Maximilians-Universität, Munich, Germany; 5 Division of Psychological and Social Medicine and Developmental Neurosciences, Faculty of Medicine, TU Dresden, Dresden, Germany; 6 Eating Disorders Research and Treatment Center, Department of Child and Adolescent Psychiatry, Faculty of Medicine, TU Dresden, Dresden, Germany

**Keywords:** Retest reliability, DSM-5, CIDI, DIA-X, Clinical interview

## Abstract

**Background:**

There is a need of comprehensive standardized diagnostic assessment tools of psychopathology that match recent changes in diagnostic classification systems, such as the 5th edition of the Diagnostic and Statistical Manual of Mental Disorders (DSM-5). Therefore, the computer-assisted DIA-X-5 was developed and its test-retest reliability was explored. The DIA-X-5 is based on the DIA-X/M-CIDI (Diagnostisches Expertensystem für psychische Störungen/Munich-Composite International Diagnostic Interview) which referred to the 4th edition of the Diagnostic and Statistical Manual of Mental Disorders (DSM-IV).

**Methods:**

A convenience sample (*N* = 60, age: 15–67) was interviewed twice with the computer-assisted DIA-X-5 interview, on average nine days apart, by trained and blinded interviewers. The DIA-X-5 is a standardized instrument for research purposes covering symptoms, syndromes and diagnoses from eleven classes of mental disorders according to the DSM-5 with matching F codes of the 10th edition of the International Classification of Diseases (ICD-10).

**Results:**

Kappa values ranged from 0.90 for post-traumatic stress disorder to 0.30 for social anxiety disorder. For age of onset and age of recency, test-retest reliability as measured by intra-class correlation was satisfying with values above 0.90 for most disorders.

**Conclusions:**

Test-retest reliability of the DIA-X-5 syndromes and diagnoses were comparable to those of previous DSM-IV/DIA-X diagnoses for most disorders. Due to low case numbers for some diagnoses, further research in larger samples is required.

## Background

The development of structured and standardized diagnostic interviews has considerably improved the reliability and validity of the assessment of mental disorders even when conducted by non-clinical (lay) interviewers [1, 2]. Within the World Health Organization (WHO) -Composite International Diagnostic Interview (CIDI; [2]) platform the DIA-X/M-CIDI (Diagnostisches Expertensystem für psychische Störungen/Munich-Composite International Diagnostic Interview; [3]) was found to possess good test-retest reliability [4] and validity [5]. It has been employed in multiple epidemiologic studies assessing mental disorders of the 4th edition of the Diagnostic and Statistical Manual of Mental Disorders (DSM-IV; [6]) and 10th edition of the International Classification of Diseases (ICD-10; [7]) in many countries [8–11].

With the publication of the fifth revision of the DSM [12], diagnostic symptom, duration, and severity criteria for several mental disorders have changed (e.g., posttraumatic stress disorder (PTSD), eating disorders). For some disorders, the classificatory position was changed (e.g. separation anxiety disorder being moved to the anxiety disorder category). For other disorders, the overall conception of the construct changed (e.g., somatoform disorders have been dismissed in favor of somatic symptom and related disorders). In addition, “new” mental disorders were defined (e.g., disruptive mood dysregulation disorder) and “specifiers” were introduced (e.g., panic attack specifier which can be added to most disorders; anxious distress specifier in bipolar and depressive disorders).

In response to the aforementioned changes, it has become necessary to provide a revised version of the DIA-X interview incorporating these changes, while maintaining the comparability to previous versions of the instrument and the implicit diagnostic algorithms to provide methodological consistency with previous studies and allow investigations into the effects of criteria changes on prevalence, onset, course and comorbidity findings. This paper presents the modification and extension process as well as test-retest reliability data of the new DIA-X-5.

## Methods

This section is structured into two major components. Firstly, the development of the DIA-X-5 interview is described, including its diagnostic coverage as well as its outer format. Secondly, the reliability is examined in a retest study for which results are presented in the current manuscript.

### Development of the DIA-X-5

The DIA-X/M-CIDI is a well-established standardized clinical interview [3] which was originally developed for clinical epidemiological purposes and served as basis for the DIA-X-5, keeping the form, the rules and conventions unchanged, whenever possible. Changes and additions were proposed, implemented and tested by a panel of experienced clinicians knowledgeable in the revision work of DSM [12] and ICD [7]. In addition, a board of external experts was invited to advise and review changes and additions to particular sections of the instrument.

### Content of the DIA-X-5: diagnostic coverage

The DIA-X-5 assesses symptoms, syndromes and diagnoses of eleven major classes of mental disorders for the lifetime and the past 12 month time frame, except for adjustment disorders and premenstrual dysphoric syndrome which are only assessed for the past 12 months. Diagnostic algorithms were reprogrammed to match recent changes in DSM-5 [12] and linked with the corresponding ICD-10 code. A complete list of DIA-X-5 diagnoses and optional modules is provided in Table 1. DSM-IV [6] algorithms are still applicable for most disorders, with exception of anorexia nervosa (amenorrhea item removed) and substance abuse (legal problem item removed). Furthermore, the DIA-X-5 includes screens for several disorders that have been added to DSM-5 ([12]; e.g. disruptive mood dysregulation disorder, premenstrual dysphoric disorder) or are related to the major diagnostic classes assessed by the DIA-X-5 (e.g. hoarding, skin picking, body dysmorphic disorder).

**Table 1:** The DIA-X-5 diagnoses (DSM-5, with corresponding ICD-10 F code) and optional modules

F-Code	Diagnosis
**A. DIA-X-5 standard diagnoses**	
*1. Neurodevelopmental disorders*	
F90.0	Attention-deficit/ hyperactivity disorder, predominantly inattentive presentation
F90.1	Attention-deficit/ hyperactivity disorder, predominantly hyperactive/impulsive presentation
F90.2	Attention-deficit/ hyperactivity disorder, combined presentation
*2. Schizophrenia spectrum and other psychotic disorders*	
F2XX	Possible psychotic disorder
F20.81	Schizophreniform disorder
F22	Delusional disorder
F23	Brief psychotic disorder
F20.9	Schizophrenia
F25.0	Schizoaffective disorder, Bipolar type
F25.1	Schizoaffective disorder, Depressive type
*3. Bipolar and related disorders* (Note: DIA-X-5 assesses symptoms for the most severe lifetime episode and the most severe past 12-month depressive/(hypo-)manic episode)	
F31.0	Bipolar I disorder, Hypomanic episode
F31.11	Bipolar I disorder, Manic episode, Mild
F31.12	Bipolar I disorder, Manic episode, Moderate
F31.13	Bipolar I disorder, Manic episode, Severe
F31.2	Bipolar I disorder, Manic episode, With psychotic features
F31.31	Bipolar I disorder, Depressed episode, Mild
F31.32	Bipolar I disorder, Depressed episode, Moderate
F31.4	Bipolar I disorder, Depressed episode, Severe
F31.5	Bipolar I disorder, Depressed episode, With psychotic features
F31.81	Bipolar II Disorder
*4. Depressive disorders*	
F32.0	Major depressive disorder, Single episode, Mild
F32.1	Major depressive disorder, Single episode, Moderate
F32.2	Major depressive disorder, Single episode, Severe
F32.3	Major depressive disorder, Single episode, With psychotic features
F33.0	Major depressive disorder, Recurrent episode, Mild
F33.1	Major depressive disorder, Recurrent episode, Moderate
F33.2	Major depressive disorder, Recurrent episode, Severe
F33.3	Major depressive disorder, Recurrent episode, With psychotic features
F34.1	Persistent depressive disorder (dysthymia)
*5. Anxiety disorders*	
F40.00	Agoraphobia
F40.10	Social anxiety disorder (social phobia)
F40.218	Specific phobia, Animal
F40.228	Specific phobia, Natural environment
F40.23X	Specific phobia, Blood-injection-injury
F40.248	Specific phobia, Situational
F40.298	Specific phobia, Other
F41.0	Panic disorder
F41.1	Generalized anxiety disorder
F93.0	Separation anxiety disorder
*6. Obsessive-compulsive and related disorders*	
F42.2	Obsessive-compulsive disorder
*7. Trauma – and stressor-related disorders*	
F43.0	Acute stress disorder
F43.10	Posttraumatic stress disorder
F43.20	Adjustment disorder, Unspecified
F43.21	Adjustment disorder with depressed mood
F43.22	Adjustment disorder with anxiety
F43.23	Adjustment disorder with mixed anxiety and depressed mood
F43.24	Adjustment disorder with disturbance of conduct
F43.25	Adjustment disorder with mixed disturbance of emotion
*8. Somatic Symptom and Related Disorders*	
F45.1	Somatic symptom disorder
F45.21	Illness anxiety disorder
*9. Feeding and Eating Disorders*	
F50.01	Anorexia nervosa, restricting type
F50.02	Anorexia nervosa, binge-eating/purging type
F50.2	Bulimia nervosa
F50.81	Binge-eating disorder
*10. Disruptive, impulse-control, and conduct disorders*	
F60.2	Antisocial personality disorder
F63.81	Intermittent explosive disorder
F91.1	Conduct disorder, childhood onset type
F91.2	Conduct Disorder, adolescent onset type
F91.3	Oppositional defiant disorder
*11. Substance-related and addictive disorders*	
F10.10	Alcohol use disorder, Mild
F10.20	Alcohol use disorder, Moderate
F10.20	Alcohol use disorder, Severe
F11.10	Opioid use disorder, Mild
F11.20	Opioid use disorder, Moderate
F11.20	Opioid use disorder, Severe
F12.10	Cannabis use disorder, Mild
F12.20	Cannabis use disorder, Moderate
F12.20	Cannabis use disorder, Severe
F13.10	Sedative, hypnotic, or anxiolytic use disorder, Mild
F13.20	Sedative, hypnotic, or anxiolytic use disorder, Moderate
F13.20	Sedative, hypnotic, or anxiolytic use disorder, Severe
F14.10	Cocaine use disorder, Mild
F14.20	Cocaine use disorder, Moderate
F14.20	Cocaine use disorder, Severe
F15.10	Amphetamine-type substance use disorder, Mild
F15.20	Amphetamine-type substance use disorder, Moderate
F15.20	Amphetamine-type substance use disorder, Severe
F16.10	Other hallucinogen use disorder, Mild
F16.20	Other hallucinogen use disorder, Moderate
F16.20	Other hallucinogen use disorder, Severe
F16.10	Phencyclidine use disorder, Mild
F16.20	Phencyclidine use disorder, Moderate
F16.20	Phencyclidine use disorder, Severe
Z72.0	Tobacco use disorder, Mild
F17.200	Tobacco use disorder, Moderate
F17.200	Tobacco use disorder, Severe
F18.10	Inhalant use disorder, Mild
F18.20	Inhalant use disorder, Moderate
F18.20	Inhalant use disorder, Severe
F19.10	Other (or unknown) substance use disorder, Mild
F19.20	Other (or unknown) substance use disorder, Moderate
F19.20	Other (or unknown) substance use disorder, Severe
**B. Other diagnostic and screening features of DIA-X-5**	
-	Substance−/Medication induced mental disorder
-	Mental disorder due to another medical condition
-	Major depressive syndrome
-	Major depressive episode
-	Manic episode
-	Hypomanic episode
-	Specifyers for mood episodes (anxious distress, mixed features, melancholic features, psychotic features, peripartum onset)
-	Panic attack specifier (for all diagnoses)
-	Disruptive mood dysregulation disorder (screening)
-	Premenstrual dysphoric syndrome (screening)
-	Hoarding disorder (screening)
-	Body dysmorphic disorder (screening)
-	Trichotillomania (Hair-pulling disorder) (screening)
-	Excoriation (Skin-picking) disorder (screening)
-	Conversion syndrome (screening)
-	Overweight/Obesity
-	Psychopathology screening for all assessed disorders (Stem screening section)
-	Family history psychopathology screening (Stem screening questions)
-	Health service utilization and treatment module
-	Embedded dimensional rating scales and disorder-related questionnaires
-	Interviewer Ratings

### Structure of the DIA-X-5: layout and outer format

For reasons of consistency the content, format, rules and style of the past DIA-X/M-CIDI were maintained. Previous data have shown that even minimal changes might influence the response behavior of the subjects [13]. Response lists were used in every section for symptoms and other items that are arranged in list format to reduce the risk of misunderstandings and increases the efficiency of the interview [14]. These lists were programmed for presentation on a tablet-computer. Thus, subjects’ responses can be directly recorded electronically and transferred wirelessly to a central data bank which eliminates the risk of coding errors during data entry. Finally, similar to the DIA-X/M-CIDI, time-related questions (e.g., age of onset) were complemented by further probe questions to improve the accuracy of estimates.

More extended changes in the DIA-X-5 include the following:

1. The structure of some disorder sections was modified to improve the interview flow given the changes in diagnostic criteria. First, in the substance use disorder sections (tobacco, alcohol, drugs), the separate assessment of abuse and dependence was omitted in favor of one comprehensive list covering all substance use disorder symptoms (without legal problems which have been omitted from DSM-5). Second, given the considerable changes surrounding somatoform disorders, this section has been changed to include questions assessing symptoms of somatic symptom disorder and omitting the previous probe questions assessing whether each individual symptom is “medically unexplained”.
2. In the section of anxiety disorders, for panic symptoms and panic disorder the order of questions was changed, given the prominent introduction of a “panic attack specifier” in DSM-5 [12], which can be coded virtually with all other diagnoses of mental disorders (except for panic disorder). Thus, the DIA-X-5 first asks for all symptoms of a panic attack before the criteria for panic disorder are enquired.
3. In disorder sections where variation in disorder symptomatology and severity are expected (e.g. mood episodes), the DIA-X-5 lifetime questions first ask for the presence of lifetime symptoms followed by questions regarding the worst lifetime episode; and finally, which of these symptoms have also been present in the past 12 months. In this way, past 12 month diagnoses assure that the full diagnostic criteria are met during the last year. Further, this additional probing allows for the derivation of partial remissions and severity coding for past year diagnoses.

### Test-retest-study: sample and procedure

A convenience sample of adolescents and adults (targeting age 14–65 years) was recruited by advertisement and in collaboration with clinical institutions. Participants were invited to participate in two diagnostic face-to-face interview sessions approximately one week apart. Upon arrival at the interview location, participants were informed about the procedures of the study and asked whether they would provide their written informed consent; for participants younger than 18 years of age, written informed assent/consent was obtained from both the adolescent and all legal guardians (e.g., both mother and father). Both interviews were conducted at the research institution (Center for Clinical Epidemiology and Longitudinal Studies at Technische Universität Dresden) or at the university hospital by two independent interviewers, i.e. the second interview was conducted blinded to the results of the first interview. Individuals were instructed to answer the questions in the second interview disregarding their answers during the first interview.

Interviews were conducted by trained clinical interviewers (psychologists as well as psychology and medical students). The 25 interviewers had a mean age of 26 years and 23 were women. Each interviewer had sufficient experience in the computerized version of the DIA-X-5 having had received a standardized multiday interviewer training and underwent a practice and certification process. Participants received payment (30€) for their participation in both interviews.

### Data analysis

Diagnostic concordance for each disorder, i.e. agreement of diagnostic results of the test (T1) and the retest interview (T2), was calculated as the relative frequency of individuals with equal disorder classification on both assessment times. The agreement for disorder categories were only calculated if the diagnostic base rate was ≥5 (at least five full cases available for analysis). Diagnostic agreement was also computed for all core DIA-X-5 (stem items) symptom questions. For diagnoses with multiple stem items, at least one of the multiple stem items had to be affirmed to compute agreement between both interviews. Calculated coefficients were Jaccard index (JI [15]), a reversed variant of the Jaccard Index (RJI) and Cohen’s kappa [16]. In the following the theoretical foundations of the coefficients JI, RJI and Cohen’s kappa are explained.

The Jaccard Index (JI [15]) is the number of individuals with a positive disorder diagnosis on both DIA-X-5 assessments divided by the number of individuals with a positive disorder diagnosis on at least one assessment. JI is an estimated lower bound for the probability that a case at one interview will be evaluated to be a case at another interview. A higher JI indicates a higher probability of re-identification of a case at another time point. JI was calculated in the following manner:

$$ JI=\frac{\# individuals\ with\ positive\ diagnosis\  on\  both\ assesments}{\# individuals\ with\ positive\ diagnosis\  on\  at\  least\  on e\  assesment\ } $$

For this study, a reversed Jaccard Index (RJI) was developed. The RJI is the number of individuals with a negative disorder diagnosis on both DIA-X-5 assessments divided by the number of individuals with negative disorder diagnosis on at least one assessment. The RJI is an estimated lower bound for the probability that a non-case at one interview will be evaluated to be a non-case at another interview. A higher RJI indicates a higher probability of re-identification of a non-case at another time point. RJI was calculated in the following manner

$$ RJI=\frac{\# individuals\ with\ negative\ diagnosis\  on\  both\ assesments}{\# individuals\ with\ negative\ diagnosis\  on\  at\  least\  on e\  assesment\ } $$

JI and RJI are easy-to-interpret and symmetric indices which mean that no pre-defined order of assessments is assumed. Thus, non-cases of the first interview (T1) that became cases at the second interview (T2) are counted like non-cases of T2 that were cases at T1, both are equally hampering the indices JI and RJI.

Cohen’s kappa [16] is the most frequently used chance-adjusted measure of agreement. It indicates the amount of difference between observed frequency of agreement and purely by chance expected frequency of agreement. Kappa ranges between − 1 (perfect disagreement) and 1 (perfect agreement), where 0 indicates chance agreement. Kappa has two well-known paradoxes [17]. First, kappa values increase whenever the true frequency of cases in a sample comes closer to 50%, regardless of the actual agreement frequency. In the present study, the frequency of cases is far below 50% for most disorders and as a consequence the reported kappa values would increase with increasing case frequencies. Second, kappa values increase with an increasing ratio of cases to non-cases from T1 to T2, regardless of the actual agreement frequency.

In the present study, for most disorders more cases emerged at T1 than at T2. Thus, also the bias-adjusted kappa (BAK; [17]) was computed. An investigation and arguments towards the use of Kappa can be found e.g. in Shrout et al. [18]. For analyses we included all disorders with at least five cases. However, Cicchetti et al. [19] suggested a case number of at least ten cases for each diagnosis. In our results all diagnoses with less than ten cases in both T1 and T2 are marked with footnotes in the tables.

Associations between time-related measures (age of onset, age of recency, persistence) were calculated as intraclass correlation coefficient (ICC). We used the one-way random-effects ICC [1] for absolute agreement ([20]; see also [21]) that corresponds to the correlation coefficient between the ratings assessed at T1 and T2 within a subject and it is also equal to the ratio of the between-subject variance of the time-related measure to the total sample variance of that time-related measure. Persistence of dysthymia was not enquired with an individual question (number of years affected by the symptoms), but was computed – given its general persistency – using the time span between age of onset and age of recency minus the longest period of time the participant indicated to feel good/OK again. For major depressive disorder with recurrent episodes the persistence corresponds to the number of years in which at least one depressive episode occurred. ICCs were calculated for all subjects who answered questions regarding the age of onset, age of recency, or persistence in both interviews, regardless of their fulfillment of the criteria for any specific diagnosis.

All analyses were conducted using Stata 14.2 [22] and confidence intervals for Cohen’s Kappa were calculated with the command ‘kapci’ [23, 24].

## Results

### Sample characteristics

The sample consisted of 60 participants, aged 15 to 67 years (*M* = 26.6 years) and included 44 women (73.3%). Most participants were attending university (58.3%) and were not married (93.3%). The mean time interval between both interviews was nine days, with a range of one to 36 days. For 46.7% of participants, the time interval was 7–9 days, for 81.7% of participants, the time interval was 4–13 days. For more details see Table 2.

**Table 2:** Sample characteristics (n = 60) and time interval between test and retest

	Number	Percent
Test-retest interval		
Average length (days)	(Mean: 9.0 / Median: 7)	
1–3 days	3	6.7
4–6 days	11	16.7
7–9 days	28	46.7
> 9 days	18	30.0
Respondents‘ characteristics		
Men	16	26.7
Women	44	73.3
Age range (15–67 years)		
Average age (years)	(Mean: 26.6 / Median: 22)	
15–21 years	29	48.3
22–30 years	19	31.7
31–50 years	7	11.7
> 50 years	5	8.3
School/employment		
School	7	11.7
University	35	58.3
Job training	2	3.3
Employed	10	16.7
Unemployed	3	5.0
Other	3	5.0
Marital status		
Unmarried	56	93.3
Educational level		
Middle	5	8.3
High	53	88.3
Other	2	3.3
History of treatment due to mental, psychosomatic or substance use problem.		
Yes	31	51.7

### Duration of the DIA-X-5

The average time to complete the DIA-X-5 excluding the sociodemographic section was 125.9 min (range 30.8–405.0 min). The duration of all sections of the interview is listed in Table 3. Of note, the applied DIA-X-5 included - for the purpose of an attached study - additional embedded questionnaires, dimensional scales as well as additional screening modules which increased the duration of most DIA-X-5 sections. With the supplementary questionnaires/ dimensional scales/ screening modules, the longest sections were those for anxiety disorder (*M =* 32.9 min) and for depressive disorders (*M* = 16.1 min). Other sections that enquire only single or less prevalent disorders were considerably shorter, such as oppositional-defiant disorder (*M =* 1.8 min) and intermittend explosive disorder (*M =* 2.0 min).

**Table 3:** Duration of the DIA-X-5 interview by section

DIA-X-5 section	Interview duration (minutes)			
	Mean	(SD)	Median	Max^b^
All sections (except non-diagnostic sections and A: Socio-demographics)	125.9	(58.7)	115.6	405.0
Section B: Tobacco use disorder	4.9	(4.9)	2.6	18.0
Section C: Somatic symptoms and related disorders (incl. Tablet module premenstrual dysphoric syndrome)	7.0	(4.7)	6.7	36.3
Section KA: Illness anxiety disorder ^a^	4.7	(4.1)	2.8	16.9
Section SA: Separation anxiety disorder ^a^	2.2	(3.2)	0.9	17.7
Section D: Anxiety disorders (panic, generalized anxiety, agoraphobia, social anxiety, specific phobia – with 5 subtypes) ^a^	32.9	(22.5)	28.5	122.4
Section E: Depressive disorders ^a^	16.1	(9.4)	16.6	44.0
Section F: Mania and Hypomania ^a^	4.1	(3.3)	2.4	15.2
Section G: Psychosis-Screening (Schizophrenia and other psychotic disorders)	4.3	(4.0)	2.6	20.6
Section H: Eating disorders	5.5	(3.7)	5.0	17.5
Section I: Alcohol use disorder ^a^	6.8	(3.8)	6.7	18.7
Section K: Obsessive-compulsive disorder (and screening for related disorders) ^a^	6.3	(4.8)	3.9	18.6
Section L: Medication and illicit drug use disorders	6.7	(6.2)	4.9	36.8
Section N: Trauma and Posttraumatic Stress Disorder	8.7	(8.7)	6.9	47.8
Section AD: Adjustment disorder	6.6	(5.0)	6.2	18.6
Section AH: Attention-deficit/ hyperactivity disorder	3.3	(2.9)	2.1	16.5
Section OD: Oppositional-defiant disorder ^a^ (incl. Tablet module for disruptive mood dysregulation disorder)	1.8	(1.8)	1.2	11.0
Section SV: Conduct / Antisocial personality disorder	3.1	(2.7)	2.3	17.9
Section IE: Intermittent explosive disorder	2.0	(2.5)	0.7	10.7
Non-diagnostic sections				
Health service utilization and treatment module	5.7	(3.3)	4.8	16.1
Interviewer observations	3.2	(3.8)	1.7	18.7
Interviewer ratings	7.1	(4.5)	5.8	26.4

^a^Section includes questionnaires/dimensional scales to assess current severity

^b^Minimum duration for all sections is about 1 min

### Diagnostic agreement for DSM-5 disorder categories

BAK values were comparable to kappa values (deviation ≤0.03) and are thus not reported in the following sections.

Test-retest reliability of DSM-5 diagnoses and diagnostic classes is displayed in Table 4.

**Table 4:** Diagnostic test-retest reliability of DSM-5 diagnostic categories with ≥5 cases for at least one interview (T1 diagnosis from test interview, T2 diagnosis from retest interview)

DSM-5 diagnosis (lifetime)	Cross-tabulation		*T1*		Jaccard Index (JI)	Agreement (%)	Cohen’s Kappa
			no	yes			
	*T2*	no	.	.	Reversed JI		(CI of Kappa)
		yes	.	.			
Any depressive disorder (major depressive disorder and dysthymia)			37	2	0.78	91.7	0.81
			3	18	0.88		(0.66–0.97)
Major depressive disorder (MDD) /			37	3	0.74	90.0	0.77
Major depressive episode^a^			3	17	0.86		(0.60–0.95)
Persistent depressive disorder (dysthymia) ^b^			51	2	0.56	93.3	0.68
			2	5	0.93		(0.38–0.97)
Any anxiety disorder (without Panic attack)			29	9	0.58	78.3	0.55
			5	18	0.69		(0.34–0.77)
Any anxiety disorder (without Social anxiety disorder)			33	6	0.64	83.3	0.65
			4	18	0.76		(0.45–0.85)
Social anxiety disorder			44	8	0.25	80.0	0.29
			4	4	0.79		(−0.02–0.59)
Any specific phobia			48	3	0.67	93.3	0.76
			1	8	0.92		(0.54–0.98)
Any specific phobia including one agoraphobic situation only			46	3	0.64	91.7	0.73
			2	9	0.90		(0.51–0.95)
Panic disorder ^b^			51	3	0.50	91.7	0.62
			2	5	0.91		(0.31–0.92)
Panic attack			39	5	0.71	90.0	0.76
			1	15	0.87		(0.59–0.94)
Panic attack specifyer (panic attack without panic disorder)			47	4	0.64	91.7	0.71
			1	8	0.90		(0.48–0.95)
Generalized anxiety disorder			47	3	0.62	91.7	0.71
			2	8	0.90		(0.47–0.95)
Obsessive-compulsive disorder ^b^			52	5	0.37	91.7	0.51
			0	3	0.91		(0.15–0.87)
Any trauma- and stressor-related disorder			43	5	0.59	88.3	0.67
			2	10	0.86		(0.44–0.89)
Post-traumatic stress disorder (PTSD) ^b^			54	0	0.83	98.3	0.90
			1	5	0.98		(0.71–1.00)
Any adjustment disorder ^c^			47	5	0.38	86.7	0.48
			2	5	0.85		(0.17–0.79)
Any somatic symptom or related disorder ^b^			49	6	0.45	90.0	0.58
			0	5	0.89		(0.29–0.87)
Somatic symptom disorder ^b^			51	5	0.44	91.7	0.58
			0	4	0.91		(0.25–0.90)
Any eating disorder			50	0	0.80	95.0	0.87
			2	8	0.96		(0.69–1.00)
Anorexia nervosa ^b^			52	0	1.00	100.0	1.00
			0	8	1.00		–
Any disruptive, impulse-control or conduct disorder ^b^			50	2	0.50	91.7	0.62
			3	5	0.91		(0.31, 0.92)
Intermittent explosive disorder ^b^			53	1	0.71	96.7	0.81
			1	5	0.96		(0.57–1.00)
Any substance use disorder			40	3	0.70	90.0	0.75
			3	14	0.87		(0.57–0.94)
Alcohol use disorder ^b^			50	2	0.60	93.3	0.71
			2	6	0.93		(0.45–0.98)
Any medication / illicit substance use disorder ^b^			53	2	0.57	95.0	0.70
			1	4	0.95		(0.38–1.00)
Cannabis use disorder ^b^			55	1	0.80	98.3	0.88
			0	4	0.98		(0.65–1.00)
Tobacco use disorder			47	2	0.77	96.7	0.84
			1	10	0.94		(0.66–1.00)
Any DSM-5 disorder			17	3	0.91	91.7	0.85
			1	39	0.81		(0.70–0.99)

^a^computed separately, no hypomanic cases and thus identical

^b^suggested criteria of at least ten positive cases is not fulfilled [19]

^c^without application of diagnostic exclusion criteria

For most disorders the JI ranged between 0.65 and 0.85. The highest JI, 1.00, was found for anorexia nervosa, second highest JI (0.89) was found for ‘any’ DSM-5 disorder. PTSD and tobacco use disorder had a JI of 0.83. Social anxiety disorder displayed the lowest JI with 0.25, followed by any obsessive compulsive disorder with 0.37.

RJIs for most disorders were 0.80 and above. Anorexia nervosa displayed highest RJI with 1.00, followed by PTSD and cannabis use disorder with 0.98. The category of any anxiety disorder (without panic attack) revealed lowest RJI with 0.69, followed by ‘any’ DSM-5 disorder and any anxiety disorder (without social anxiety disorder) with an RJI of 0.76 each.

Highest agreement with 100.0% was found anorexia nervosa, followed by a 98.3% agreement rate for PTSD and cannabis use disorder. Any DSM-5 disorder had an agreement of 91.7%. The categories of any anxiety disorder (without panic attack) and social anxiety disorder revealed the lowest agreement with 78.3 and 80.0% respectively. However, most diagnostic categories displayed an agreement of 90% and above. Most Cohen’s kappa values ranged between 0.70–0.85, with anorexia nervosa displaying the highest kappa (1.00), followed by PTSD (0.90), tobacco use disorder (0.89) and cannabis use disorder (0.88). The diagnosis of ‘any’ DSM-5 disorder had a kappa of 0.81. Cohens Kappa was lowest for social anxiety disorder (0.29), followed by any obsessive-compulsive disorder (0.51) and any anxiety disorder without panic attack (0.55). For discordant cases it was also tested whether for those disorders with all fulfilled criteria in one interview, at least the respective stem question was endorsed in the other interview. Of the two single disorders with low kappa, the respective stem question was always endorsed for social anxiety disorder. However, for obsessive-compulsive disorder (OCD) two out of three cases did not endorse the stem question in one interview although they fulfilled all criteria for a diagnosis in the other interview.

### Diagnostic agreement on core DIA-X-5 symptoms

Diagnostic agreement for the core DIA-X-5 stem items are shown in Table 5. Separation anxiety disorder displayed the lowest JI (0.33), whereas any drug use disorder - illicit drug consume (*JI =* 0.94) and major depressive disorder (*JI =* 0.91) displayed the highest JIs. For most disorders, JIs ranged between 0.65 and 0.85. RJI was the highest for any drug use disorder - illicit drug consume (0.98), followed by tobacco use disorder – symptom item (0.95) and conduct disorder (0.94). Medication use – consume item had the lowest RJI with 0.64. For most disorders the RJI was 0.85 and above.

**Table 5:** Test-retest reliability of the DIA-X-5 core (stem) items

Stem Items for diagnosis (lifetime)	Cross-tabulation		*T1*		Jaccard Index (JI)	Agreement (%)	Cohen’s Kappa
			no	yes			
	*T2*	no	.	.	Reversed JI		(CI of Kappa)
		yes	.	.			
Neurodevelopmental disorders							
Attention-deficit/ hyperactivity disorder			30	11	0.57	78.3	0.56
			2	17	0.70		(0.35–0.76)
Schizophrenia spectrum and other psychotic disorders							
Possible psychotic disorder			24	6	0.83	90.0	0.80
			0	30	0.80		(0.65–0.95)
Bipolar and related disorders							
Manic / hypomanic episode			29	11	0.55	76.7	0.52
			3	17	0.67		(0.31–0.73)
Depressive disorders							
Major depressive disorders			17	4	0.91	93.3	0.85
			0	39	0.81		(0.70–0.99)
Persistent depressive disorder (dysthymia)			33	10	0.63	83.3	0.65
			0	17	0.77		(0.47–0.84)
Disruptive mood dysregulation disorder^a, b^			51	2	0.44	91.7	0.57
			3	4	0.91		(0.23–0.91)
Anxiety disorders							
Agoraphobia			46	5	0.64	91.7	0.73
			0	9	0.90		(0.52–0.95)
Social anxiety disorder			21	1	0.87	91.7	0.83
			4	34	0.81		(0.68–0.97)
Specific phobia			40	2	0.80	93.3	0.84
			2	16	0.91		(0.69–0.99)
Panic disorder			31	5	0.76	88.3	0.76
			2	22	0.82		(0.60–0.93)
Generalized anxiety disorder			30	3	0.87	93.3	0.87
			1	26	0.88		(0.74–0.99)
Separation anxiety disorder ^b^			48	6	0.33	86.7	0.43
			2	4	0.86		(0.11–0.75)
Obsessive-compulsive and related disorders							
Obsessive-compulsive disorder			30	3	0.87	93.3	0.87
			1	26	0.88		(0.74–0.99)
Trauma- and stress-related disorders							
Post-traumatic stress disorder (PTSD) / Acute stress disorder			24	3	0.78	86.7	0.73
			5	28	0.75		(0.56–0.90)
Adjustment disorder			16	4	0.82	86.7	0.70
			4	36	0.67		(0.51–0.89)
Somatic symptom and related disorders							
Somatic symptom disorder			11	3	0.90	91.7	0.76
			2	44	0.69		(0.56–0.96)
Illness anxiety disorder			40	4	0.75	91.7	0.80
			1	15	0.89		(0.63–0.97)
Eating disorders							
Weight loss stem item			18	6	0.81	86.7	0.71
			2	34	0.69		(0.53–0.90)
Binge eating stem item			47	2	0.77	95.0	0.84
			1	10	0.94		(0.66–1.00)
Disruptive, impulse-control, and conduct disorders							
Intermittent explosive disorder			43	5	0.71	91.7	0.77
			0	12	0.90		(0.59–0.96)
Conduct or antisocial personality disorder			45	3	0.80	95.0	0.86
			0	12	0.94		(0.70–1.00)
Oppositional defiant disorder			41	6	0.53	85.0	0.59
			3	10	0.82		(0.35–0.83)
Substance-related and addictive disorders							
Alcohol use disorder – consume stem item			15	1	0.87	90.0	0.76
			5	39	0.71		(0.59–0.94)
Alcohol use disorder – symptom stem item			40	2	0.70	90.0	0.75
			4	14	0.87		(0.57–0.94)
Medication use – consume stem item			29	7	0.48	73.3	0.44
			9	15	0.64		(0.20–0.67)
Any drug use disorder – illicit drug consume stem question			43	0	0.94	98.3	0.96
			1	16	0.98		(0.88–1.00)
Any drug use disorder – symptom stem item			44	6	0.50	86.7	0.59
			2	8	0.85		(0.33–0.84)
Tobacco use disorder – smoking stem item			35	3	0.88	95.0	0.90
			0	22	0.92		(0.78–1.00)
Tobacco use disorder – symptom stem item			42	2	0.89	96.7	0.92
			0	16	0.95		(0.81–1.00)

^a^Partly assessed by embedded questionnaires

^b^suggested criteria of at least ten positive cases is not fulfilled [19]

Agreement was highest for any drug use disorder – illicit drug consume (98.3%) and tobacco use disorder – symptom item (96.7%). Medication use – consume item had the lowest agreement rate of 73.3%. For most disorders, agreement was 80% and above.

Kappa values were highest for any drug use disorder – illicit drug consume (0.96) and were also high for tobacco use disorder – symptom item (0.92). Separation anxiety disorder had the lowest kappa value with 0.43, followed by medication use – consume item 0.44. Stem items of most disorders had kappa values between 0.70 and 0.85.

### Agreement on time-related questions

ICCs for age of onset, age of recency, and persistence are displayed in Table 6. ICCs could not be computed for manic episodes, attention deficit hyperactivity disorder (ADHD) with predominantly hyperactive/impulsive presentation and disruptive mood dysregulation disorder because of too few cases.

**Table 6:** Reliability of age of onset, age of recency, and persistence as measured by the intra-class correlation coefficient (ICC)

Variables by diagnostic category	Age of Onset		Age of Recency		Persistence	
	N (N_mis_)	ICC	N (N_mis_)	ICC	N (N_mis_)	ICC
Neurodevelopmental disorders						
ADHD, predominantly inattentive presentation	9 (5)	0.92	9 (6)	0.99	9 (6)	0.66
ADHD, predominantly hyperactive/impulsive presentation^a^	–	–	–	–	–	–
Schizophrenia spectrum and other psychotic disorders						
Possible psychotic disorder	12 (8)	0.93	12 (8)	0.99	12 (8)	0.20
Bipolar and related disorders						
Manic episode^a^	–	–	–	–	–	–
Depressive disorders						
Major depressive disorder, Single episode	7 (10)	0.99	7 (10)	0.99	7 (10)	0.90
Major depressive disorder, Recurrent episode	25 (9)	0.40	38 (5)	0.94	20 (14)	0.45
Persistent depressive disorder (dysthymia)	16 (13)	0.94	16 (13)	0.98	16 (13)	0.61
Disruptive mood dysregulation disorder ^a^	–	–	–	–	–	–
Anxiety disorders						
Agoraphobia	8 (6)	0.91	9 (5)	0.96	1 (3)	–
Social anxiety disorder	31 (8)	0.73	34 (5)	0.98	15 (13)	0.81
Specific phobia, Animal type	14 (6)	0.83	15 (5)	0.99	10 (3)	0.50
Specific phobia, Natural environment type	11 (8)	0.84	12 (7)	0.95	7 (4)	0.94
Specific phobia, Blood-injection-injury type	10 (10)	0.53	11 (9)	0.90	6 (6)	0.80
Specific phobia, Situational/Other type ^b^	4 (7)	−0.74	6 (5)	0.61	4 (5)	0.96
Panic disorder	9 (4)	0.99	22 (7)	0.99	9 (4)	0.81
Generalized anxiety disorder	26 (4)	0.63	26 (4)	0.95	25 (5)	0.25
Separation anxiety disorder	4 (6)	0.96	4 (6)	0.98	4 (6)	0.65
Obsessive-compulsive and related disorders						
Obsessive-compulsive disorder, Thoughts	14 (10)	0.85	17 (7)	0.94	10 (8)	0.54
Obsessive-compulsive disorder, Behavior	16 (8)	0.84	22 (2)	1.00	13 (9)	0.85
Trauma- and stressor related disorders						
Post-traumatic stress disorder	12 (11)	0.99	12 (11)	0.97	12 (11)	0.81
Somatic symptom and related disorders						
Somatic symptom disorder	44 (5)	0.84	44 (5)	0.98	18 (12)	0.16
Illness anxiety disorder	9 (4)	0.91	9 (4)	0.93	9 (4)	0.34
Feeding and eating disorders						
Anorexia nervosa	11 (3)	0.74	11 (3)	0.99	13 (4)	0.84
Bulimia nervosa	4 (3)	0.91	15 (5)	0.94	13 (6)	0.76
Binge eating disorder	4 (3)	0.91	4 (3)	0.99	4 (3)	0.86
Disruptive, impulse-control, and conduct disorders						
Antisocial personality disorder	4 (0)	0.99	9 (2)	0.77	9 (2)	0.91
Intermittent explosive disorder	11 (5)	0.85	12 (5)	0.81	11 (5)	0.64
Conduct disorder, childhood onset type	7 (3)	0.93	7 (3)	0.91	6 (3)	0.64
Oppositional defiant disorder	4 (8)	0.93	4 (8)	0.79	6 (6)	0.65
Substance-related and addictive disorders						
Alcohol use disorder	13 (7)	0.92	14 (6)	0.87	13 (7)	0.43
Tobacco use disorder	16 (2)	0.79	16 (2)	0.99	16 (2)	0.81

N = participants with time-related measures in both interviews; N_mis_ = participants with time-relevant measures in only one interview

^a^No sufficient number of cases

^b^Based on four cases including one outlier

For most diagnoses, ICCs for age of onset were 0.90 and above. ICC for age of onset was highest for PTSD, panic disorder, single major depressive disorder and antisocial personality disorder with 0.99. ICC for onset was lowest for specific phobia with − 0.74 (situational/other type) and also low for major depressive disorder with recurrent episodes (0.40).

ICCs of age of recency were above 0.90 for most disorders. An ICC of 1.00 was found for recency of obsessive-compulsive disorder (behavior). Also, ICCs were high with 0.99 for tobacco use disorder, anorexia nervosa, binge eating disorder, panic disorder, specific phobia (animal type), single major depressive episode, possible psychotic disorder and ADHD (inattentive presentation). The lowest ICC for recency was shown by specific phobia (situational/other type) with 0.61.

Most disorders revealed ICCs for persistence between 0.55 and 0.85. Persistence reliability was highest for specific phobia (situational/other type) with 0.96, followed by specific phobia (natural environment type) with 0.94. Lowest ICC for persistence was revealed by somatic symptom disorder with 0.16, followed by possible psychotic disorder with 0.20.

## Discussion

The standardized assessment of symptoms, syndromes and diagnoses of mental disorders is essential for estimating the prevalence, onset, and course of mental disorders and determining their risk factors in epidemiologic research. Case definition in clinical and experimental studies also relies on reliable diagnoses. The standardized and fully computerized DIA-X-5 reveals good test-retest reliability for most DSM-5 diagnoses, stem items and time-related information in adolescents and adults.

### Test-retest reliability of diagnoses and stem items of the DIA-X-5

Although most of the DIA-X-5 diagnoses showed good to very good test-retest reliability, some diagnoses showed relatively low reliability. For these diagnoses we examined in more detail the response patterns on the level of diagnostic criteria and individual questions.

The summary category of any anxiety disorder (without panic attack) reveals relatively low reliability because of low reliability indices for few specific anxiety diagnoses. For panic disorder, comparing each separate diagnostic criterion, there was no specific response pattern which changed from the first to the second interview. However, a change in the order of questions in this section may have affected subjects’ overall response behavior. In the DIA-X/M-CIDI, the panic attack stem question was followed by panic disorder questions only after which the panic attack symptoms were assessed. This order changed in the DIA-X-5, probing the panic attack symptoms before the panic disorder criteria, due to the prominent role of the panic attack specifier in DSM-5 [12]. Reliability for the panic stem item was good, as was the reliability for panic attack.

For social anxiety disorder, participants’ responses varied particularly for avoidance (5 of 12 discordant cases) and duration of anxiety (6 out of 12 discordant cases). Social anxiety disorder consists of a long criterion list and 9 out of 12 discordant cases were discordant only in one criterion. Agoraphobia and separation anxiety disorder did have a low overall number of cases in this study hampering reliability estimates.

For obsessive-compulsive disorder, different criteria for thoughts and behavior revealed divergent responses patterns between both interviews. For obsessive thoughts, mainly the response to the A criterion changed between both interviews. For compulsive behavior, the list of items/behaviors was expanded in the DIA-X-5 to also probe for OCD-related syndromes including nail biting, hair pulling, skin picking, and mirror checking. Although the mere presence of these symptoms was not counted toward the standard diagnosis of OCD, their inclusion may have affected the responses for OCD, given that symptoms such as nail biting were quite prevalent in the sample. Most discordant OCD-cases were due to the B criterion – referring to distress/impairment (mostly rated “some” in the second interview instead of “much”).

As already noted, the stem items of most diagnoses showed high reliability, even for the disorders for which relatively low reliability indices were found on the diagnostic level. It should be noted though that the stem question for the use of legal drugs, i.e. medication, reveals low reliability indices. This might depend on the type of listed substances and the broad open category of “other medications”. Unfortunately, no cases of medication use disorder were found in the current study, not allowing to test whether retest-reliability for medication would be higher on the diagnostic level.

When comparing the retest-reliability of diagnoses and stem items of the DIA-X-5 with previous results of the DIA-X/M-CIDI [4], kappa values on the diagnostic level are similar for depressive disorders, alcohol and illicit drug use disorder as well as for any DSM disorder. For PTSD, tobacco use disorder and any eating disorder, the DIA-X-5 reveals higher kappa values (kappa deviating at least 0.1), whereas the DIA-X/M-CIDI revealed higher kappa values for obsessive compulsive disorder and any somatoform disorder (relative to somatic symptom disorder). Mixed results were found for anxiety disorders; the DIA-X-5 had higher kappa values for panic attack and generalized anxiety disorder, the DIA-X/M-CIDI had higher kappa values for most anxiety disorder categories, such as any anxiety disorder, social anxiety disorder and panic disorder. However, most of these differences equal out when including kappa values for the stem items which reveal comparable kappa values for panic disorder, social anxiety disorder, agoraphobia, specific phobia, major depressive disorder, eating disorder and PTSD between DIA-X-5 and DIA-X/M-CIDI. However, relevant differences in kappa still remain for the stem items of generalized anxiety disorder (GAD) and OCD – with higher kappa values in the DIA-X-5 – and for dysthymia and manic/hypomanic episode – with higher kappa values for the DIA-X/M-CIDI. The differences between DIA-X/M-CIDI and DIA-X-5 likely depend on the available number of cases, which was higher in the DIA-X/M-CIDI retest study resulting in higher kappa values (first kappa paradox). As previously mentioned, the change in the order of questions for panic disorder might have decreased the kappa values for panic disorder in the DIA-X-5, in comparison to the DIA-X/M-CIDI.

### Test-retest reliability of time-related questions

High test-retest reliability was found for the age of onset and age of recency questions in the DIA-X-5; the persistence questions generally revealed slightly lower reliability. Compared to the previous DIA-X/M-CIDI, the ICCs for age of onset in the DIA-X-5 were either similar or somewhat higher for most disorders. Substantially higher ICCs for age of onset were found in the DIA-X/M-CIDI, however, for most specific phobia subtypes, which could be due to the greater number of specific phobia cases in the DIA-X/M-CIDI retest study. In the current study low ICC in age of onset resulted from an overall small number of cases. For the specific phobia subtype, this was combined with an outlier who reported extremely different age of onsets in both interviews. Low ICC’s for age of recency in disruptive mood dysregulation disorder also may be due to few overall cases.

Persistence revealed a stronger variability than the other two time related measures. For specific phobias (situational/natural environment/other type), participants reported diverging onset/recency, most likely because these disorders often manifest early in development and take a waxing and waning course. However, overall persistence - meaning the overall number of years affected - is remembered similarly in both interviews. For illness anxiety and somatic symptom disorder a slow development and varying intensity levels can be assumed leading to difficulties in estimating persistence in terms of number of years affected. For separation anxiety disorder there were too few cases in this study to make reliable conclusions.

### Limitations

This test-retest study has limitations: First, the number of subjects assessed is at the lower bound for a test-retest reliability study, which principally affects the reliability estimates for conditions *less frequently* diagnosed in the sample. However, previous studies on retest reliability of structured clinical interviews included samples of comparable and even lower size with a range of 60 to 43 participants for the M-CIDI [4], the Structured Clinical Interview for DSM-5 disorders (DSM-5 SCID [25]) and the Spanish version of the Kiddie Schedule for Affective Disorders and Schizophrenia present and lifetime version DSM-5 (K-SADS-PL-5 [26]). Second, the test-retest interval varies between one and 36 days. Although an average retest interval of nine days is appropriate considering the variability for example depressive symptoms, shorter intervals (below 7 days) could increase kappa estimates. Third, a convenience sample was recruited for this study. Thus, the sample is community based and therefore useful for an instrument which is designed for representative studies. Fourth, the DIA-X-5 version applied in this study included a range of additional nested questionnaires, lists and screening modules which increased the length of the DIA-X-5 sections. This may have affected the response behavior of the participants. The relatively high agreements of the stem questions, however, argue against a systematic response behavior bias. Finally, the reliability coefficients of some disorders are at a lower bound. Those include panic disorder, social anxiety disorder and obsessive-compulsive disorder. In addition, some disorders reveal a fair Cohens kappa CI higher than 0.40 but do not meet the suggested criteria of ten cases [19], those are PTSD, anorexia nervosa, intermittent explosive disorder, and cannabis use disorder. Some reveal a kappa lower CI < 0.40 or a kappa CI range > .50, those disorders include persistent depressive disorder, panic disorder, obsessive compulsive disorder, any adjustment disorder, somatic symptom disorder, and alcohol use disorder. Concerning the diagnoses of these disorders the DIA-X-5 should be used with caution. Of note, reliability of stem items of these disorders is sufficient.

## Conclusions

The DIA-X-5 is an extended, modified version of the DIA-X/M-CIDI. For the mental disorders with sufficient case numbers and thus analyzed in the present study, the DIA-X-5 reliably assesses symptoms, syndromes and diagnoses according to DSM-5 including their onset, recency, and persistence, when applied face-to-face by trained interviewers. These disorders are from DSM-5 sections containing the most prevalent mental disorders: depressive, anxiety, trauma-related, somatic symptom, eating, substance use disorders, and disruptive, impulse control or conduct disorders. The limited sample size calls for additional studies replicating the findings and allowing for more reliable conclusions with regard to less prevalent disorders. Finally, this study focused on the reliability of the DIA-X-5. However, its validity needs to be evaluated in a separate study.

## Data Availability

The datasets used and/or analysed during the current study are available from the corresponding author on reasonable request.

## Abbreviations

DIA-X: Diagnostisches Expertensystem für Psychische Störungen
M-CIDI: Munich-Composite International Diagnostic Interview
DSM: Diagnostic and Statistical Manual of Mental Disorders
ICD: International Classification of Diseases
WHO: World Health Organization
PTSD: Post traumatic stress disorder
JI: Jaccard Index
RJI: Reversed Jaccard Index
BAK: Bias-adjusted kappa
ICC: Intraclass correlation coefficient
OCD: Obsessive-compulsive disorder
ADHD: Attention deficit hyperactivity disorder
GAD: Generalized anxiety disorder
SCID: Structured Clinical Interview for DSM Disorders
K-SADS-PL-5: Spanish version of the kiddie schedule for affective disorders and schizophrenia present and lifetime version DSM-5
