# Correction to: Test-retest reliability of the computer-assisted DIA-X-5 interview for mental disorders

**DOI:** 10.1186/s12888-020-02762-2

**Published:** 2020-07-09

**Authors:** Jana Hoyer, Catharina Voss, Jens Strehle, John Venz, Lars Pieper, Hans-Ulrich Wittchen, Stefan Ehrlich, Katja Beesdo-Baum

**Affiliations:** 1 Institute of Clinical Psychology and Psychotherapy, Technische Universität Dresden, Dresden, Germany; 2 Behavioral Epidemiology, Faculty of Psychology, Technische Universität Dresden, Dresden, Germany; 3 Center for Clinical Epidemiology and Longitudinal Studies, Technische Universität Dresden, Dresden, Germany; 4 Department of Psychiatry & Psychotherapy, Ludwig-Maximilians-Universität, Munich, Germany; 5 Division of Psychological and Social Medicine and Developmental Neurosciences, Faculty of Medicine, TU Dresden, Dresden, Germany; 6 Eating Disorders Research and Treatment Center, Deartment of Child and Adolescent Psychiatry, Faculty of Medicine, TU Dresden, Dresden, Germany

**Correction to: BMC Psychiatry (2020) 20:280**

**https://doi.org/10.1186/s12888-020-02653-6**

Following the publication of the original article [1], multiple errors were identified in the Results Text of the Abstract and the Diagnostic agreement for DSM-5 disorder categories section.

The text has been changed to correspond to the correct results reported in Table 4 and are highlighted in bold typeface.

Abstract - Results:

Kappa values ranged from 0.90 for post-traumatic stress disorder to **0.29** for social anxiety disorder.

Main text - Diagnostic agreement for DSM-5 disorder categories:

The highest JI, 1.00, was found for anorexia nervosa, second highest JI **(0.91)** was found for ‘any’ DSM-5 disorder. PTSD had a JI of 0.83.

The category of any anxiety disorder (without panic attack) revealed lowest RJI with 0.69, followed by **social anxiety** disorder **with an RJI of 0.79; the RJI for** any anxiety disorder (without social anxiety disorder) was 0.76.

Most Cohen’s kappa values ranged between 0.70 – 0.85, with anorexia nervosa displaying the highest kappa (1.00), followed by PTSD (0.90) and cannabis use disorder (0.88). The diagnosis of ‘any’ DSM-5 disorder had a kappa of **0.85**.
